# Naive stem cell blastocyst model captures human embryo lineage segregation

**DOI:** 10.1016/j.stem.2021.04.031

**Published:** 2021-06-03

**Authors:** Ayaka Yanagida, Daniel Spindlow, Jennifer Nichols, Anish Dattani, Austin Smith, Ge Guo

**Affiliations:** 1 Living Systems Institute, University of Exeter, Stocker Road, Exeter EX4 4QD, UK; 2 Wellcome-MRC Cambridge Stem Cell Institute, University of Cambridge, Puddicombe Way, Cambridge CB2 0AW, UK; 3 Department of Physiology, Development and Neuroscience, University of Cambridge, Cambridge CB2 1GA, UK

**Keywords:** embryonic stem cells, pluripotency, human embryo, blastocyst, epiblast, hypoblast, trophoblast, lineage segregation, self-organization

## Abstract

Human naive pluripotent cells can differentiate into extraembryonic trophectoderm and hypoblast. Here we describe a human embryo model (blastoid) generated by self-organization. Brief induction of trophectoderm leads to formation of blastocyst-like structures within 3 days. Blastoids are composed of three tissue layers displaying exclusive lineage markers, mimicking the natural blastocyst. Single-cell transcriptome analyses confirm segregation of trophectoderm, hypoblast, and epiblast with high fidelity to the human embryo. This versatile and scalable system provides a robust experimental model for human embryo research.

## Highlights

• Generation of human blastoids with structure and composition similar to the embryo
• Segregation of trophectoderm, hypoblast, and naive epiblast lineages
• Transcriptome fidelity with the embryo
• Simple, efficient, and scalable procedure

## Graphical abstract

**Figure:**
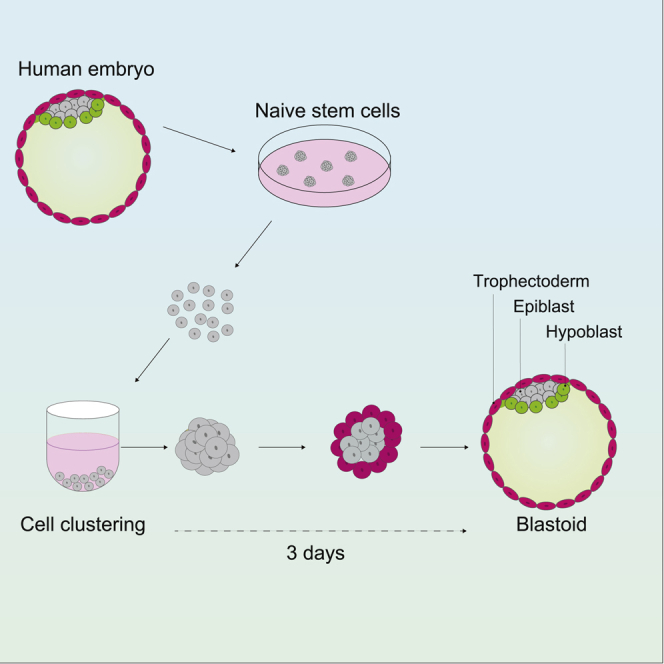

## Teaser

Reliable stem cell models of the early embryo would open new approaches to studying human development and infertility. Yanagida and colleagues used naive stem cells to generate human blastocyst-like structures comprising the three founding tissue layers for the extraembryonic membranes and the embryo.

## Introduction

Natural development of the human embryo is challenging to study *in vivo* and few embryos are available for research *in vitro*. Scientists have therefore relied heavily on observations and experiments in other mammals, in particular mice. However, although embryology unfolds according to a similar overall program in all mammals, there are many distinctions between species. Even the first morphogenetic process—formation of the blastocyst—is regulated differently in human and mouse (Guo et al., 2021; Roode et al., 2012; Rossant, 2018). The blastocyst is a landmark of eutherian development that is essential for uterine implantation. Blastocyst formation initiates with delamination of epithelial trophectoderm cells on the surface of the unspecified morula. The trophectoderm forms a fluid-filled cavity and the internal inner cell mass (ICM) cells differentiate into two further lineages, epiblast and hypoblast (also known as primitive endoderm). The mature blastocyst formed by embryonic day 6 (E6) in human is a simple cavitated structure comprising three topologically and molecularly segregated lineages, each of which is critical for further development.

In recent provocative research, blastocyst-like structures termed blastoids have been generated from mouse stem cells (Rivron et al., 2018; Sozen et al., 2019). Approaches have subsequently been devised to manufacture representations of the human blastocyst (Liu et al., 2021; Yu et al., 2021). To be a useful model, however, blastoids must accurately recapitulate the cellular organization and lineage composition of the natural human blastocyst (Rossant and Tam, 2021).

Here we investigated the potential of human naive stem cells (Bredenkamp et al., 2019b; Guo et al., 2016; Takashima et al., 2014) to generate blastoids that show high fidelity to the human embryo and fulfil key criteria for an experimental model system.

## Results

Recently we found that human naive pluripotent stem cells can generate mixed cultures comprising the three founding lineages of the blastocyst (Guo et al., 2021). Trophectoderm differentiation is initiated by inhibition of ERK and NODAL signaling using small molecules PD0325901 and A83-01 (PD+A83). We also observed that treatment with PD+A83 in suspension resulted in appearance of epithelial cysts expressing the trophectoderm reporter *GATA3:mKO2*. We noticed that cysts often contained inner cells that were initially mKO2 negative but became positive upon continued exposure to PD+A83. These findings suggested the possibility of generating blastocyst-like structures from naive stem cells by modulating PD+A83 treatment.

**Figure 1:**
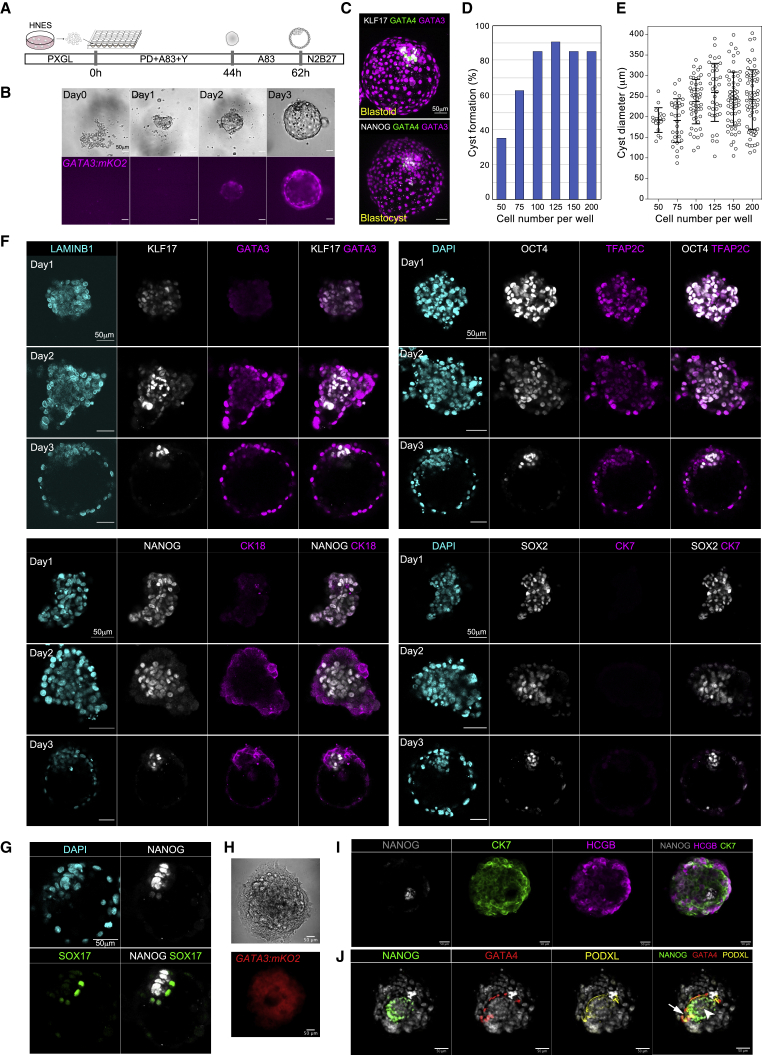
Formation of model human blastocysts (blastoids) (A) Schematic of procedure. (B) Phase contrast and florescence images showing formation of a *GATA3:mKO2* positive cyst. (C) z-projections of a day 3 blastoid and a human late blastocyst (E7) stained for epiblast (KLF17, NANOG), hypoblast (GATA4), and trophectoderm (GATA3) markers. (D) Quantification of cavitated mKO2-positive cyst formation related to cell number seeded. (E) Quantification of diameter of cysts as in (D). Error bars are SD. (F) Immunofluorescence staining for epiblast and trophectoderm markers during blastoid formation. (G) Immunofluorescence staining for hypoblast marker SOX17 in day 3 blastoid. (H) Phase contrast and fluorescence images of GATA3:mKO blastoid outgrowth after 4 days. (I) z-projections of immunofluorescence staining of day 4 outgrowth stained for differentiated trophoblast (CK7) and syncytiotrophoblast (HCGB) markers and NANOG. (J) z-projections of immunofluorescence staining of day 4 outgrowth stained for PODXL, NANOG, GATA4, and DAPI. Arrows point to cavities (see Video S1). Scale bars in all images are 50 μm.

We set up trials in non-adherent 96-well U-bottom plates using *GATA3:mKO2* to monitor the formation and distribution of trophectoderm cells. Cells were dissociated and seeded in PD+A83 plus Rho-associated kinase inhibitor Y-27632 to aid survival. The day after seeding, loose aggregates formed. GATA3:mKO2 expression was apparent in surface cells on day 2 and cavitation initiated. At the end of day 2, we exchanged the medium to culture in A83 only. On day 3 many of the aggregates formed expanded cysts of *GATA3:mKO2*-positive cells with a GATA3-negative inner population (Figures 1A and 1B). We detected non-overlapping expression of naive epiblast (KLF17) and hypoblast (GATA4) markers in the inner population, as in the mature human blastocyst (Figure 1C).

The initial cell seeding number influenced the efficiency of cyst formation. Between 100 and 150 cells, we obtained single cavitated structures in more than 80% of wells (Figure 1D). Seeding fewer cells gave mainly compact aggregates of GATA3-positive cells (Figures S1A–S1C) and at higher seeding density multiple cysts formed per well. The size of cysts varied, with an average diameter of about 250 μm, similar to the late human blastocyst (Figures 1E and S1A–S1C). Blastocyst-like structures maintained integrity on day 4 after removal of A83.

By immunofluorescence staining we characterized expression of trophectoderm and epiblast markers in day 3 cysts (Figure 1F). GATA3 protein was readily detected from day 2 and localized in the outer layer together with the epithelial protein keratin 18 (CK18). On day 3, GATA3 was prominent in nuclei of all outer cells and absent from inside cells. In contrast, epiblast markers KLF17, NANOG, OCT4, and SOX2 that are ubiquitous in naive pluripotent cells (Bredenkamp et al., 2019b) were confined to inner cells by day 3. Expression of trophectoderm and epiblast markers was generally exclusive, as observed in the human mature blastocyst. Notably the core pluripotency factor OCT4 was restricted to inner cells that were all GATA3 negative. OCT4 can be detected in trophectoderm in the early human blastocyst (E5), but it is confined to the ICM by the fully expanded E6 blastocyst (Deglincerti et al., 2016; Niakan and Eggan, 2013; Shahbazi et al., 2016). TFAP2C (AP2γ) is known as a trophoblast marker in mouse but in human is also expressed in naive epiblast and naive stem cells (Pastor et al., 2018). We detected moderate expression of TFAP2C in inner cells with upregulation in the outer epithelium. Presence of KLF17 in inner cells indicated that they remain in the pre-implantation stage of naive pluripotency (Blakeley et al., 2015; Boroviak et al., 2015; Rostovskaya et al., 2019). Finally, barely detectable keratin 7 (CK7) is consistent with pre-implantation trophectoderm in contrast to post-implantation cytotrophoblast (Deglincerti et al., 2016).

By E6 the human ICM has segregated into naive epiblast and hypoblast (Niakan and Eggan, 2013; Petropoulos et al., 2016; Roode et al., 2012; Stirparo et al., 2018). We assessed presence of hypoblast in day 3 cysts. We detected GATA4-positive cells in 36 of 44 cysts (Figures S1D and S1E). The number of GATA4-positive cells varied (Figure S1F), as observed for hypoblast cells in human embryos (Niakan and Eggan, 2013; Roode et al., 2012). We also detected expression of hypoblast markers SOX17 and OTX2 (Boroviak et al., 2018) in cells that typically appeared to underly the SOX2-positive epiblast (Figures 1G and S1H).

We examined cyst formation from other embryo-derived (HNES2, HNES3), chemically reset (cR_Shef6) (Guo et al., 2017), and directly reprogrammed (niPSC_HDF75) (Bredenkamp et al., 2019b) naive stem cells. We obtained expanded blastocyst-like structures within 3 days in all cases although efficiency ranged from around 30% for cR-Shef6 to greater than 80% for niPSC_HDF75 (Figures S1G). By day 4 all three lineages were consistently present (Figures S1I and S1J).

Overall, the localization of epiblast, hypoblast, and trophectoderm markers indicates that the cysts attain a developmental stage comparable to the late human blastocyst (E6/7) in which the three founding embryo lineages are fully segregated. We therefore termed these cysts human blastoids.

After day 4 blastoid cavities collapsed, similar to human blastocysts by the end of day E7 in suspension (Shahbazi et al., 2016). When transferred to Geltrex-coated dishes, blastoids attached and proliferated to produce large outgrowths of *GATA3:mKO2*-positive cells (Figure 1H). Outgrowing cells displayed cytokeratin 7 (CK7) and included regions expressing the syncytiotrophoblast marker human β chorionic gonadotrophin (hCGB) (Figure 1I), similar to human blastocyst outgrowths (Deglincerti et al., 2016). In a portion of the cultures, a compact area of small cells was apparent that expressed the epiblast marker NANOG with adjacent cells expressing GATA4 (Figures 1I and 1J). In these regions we detected podocalyxin (PODXL) stained lumen structures (Bedzhov and Zernicka-Goetz, 2014; Shahbazi et al., 2016), suggestive of emergent yolk sac cavities and amniotic cavities (Figure 1J; Video S1). The cavities were bounded by NANOG-positive cells and embedded within extensive trophoblast outgrowths. Conventional hPSCs can self-organize to form lumens and amnion-like cavities (Taniguchi et al., 2015; Zheng et al., 2019). Integration with differentiating trophoblast is therefore necessary to assert progression from the blastoid ICM. Nonetheless, more comprehensive analyses are required to make definitive statements regarding the emergence of these structures and their relationship to development of the bilaminar embryonic disc.

Video S1. Z series confocal scanning through blastoid outgrowth shown in Figure 1J, related to Figure 1 Cavities indicated by arrows on the section in Figure 1J can be seen in 3D in the Z series. White, DAPI; green, NANOG; red, GATA4; yellow, PODXL.

**Figure 2:**
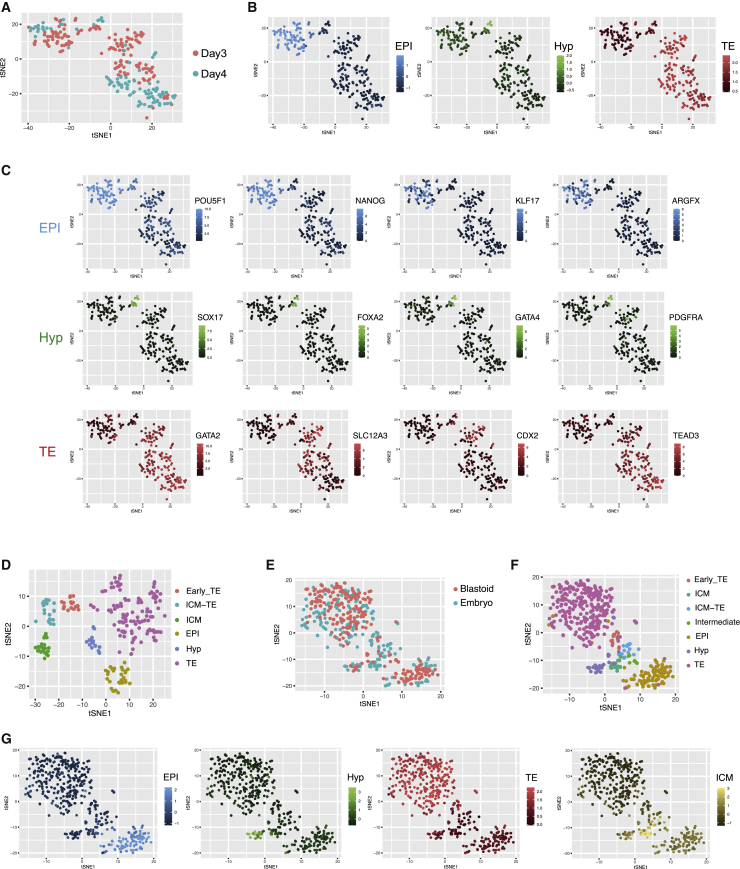
Single-cell transcriptome analysis (A) t-distributed stochastic neighbor embedding (tSNE) of day 3 and day 4 blastoid cells. (B) tSNE plot in (A) showing averaged expression scores of classifier genes (Table S1) enriched in human E6/7 embryo lineages, epiblast (EPI), hypoblast (Hyp), and trophectoderm (TE). (C) Expression (FPKM) of selected lineage markers on tSNE plot in (A). (D) tSNE analysis of human embryos at E5, E6, E7 with lineage assignment by marker profile. ICM_TE denotes ambiguous ICM and TE identity. (E) Integrated tSNE plot for blastoid and embryo cells. (F) tSNE plot in (E) colored to show cell lineage assignments from Figures S1A and S1D. (G) tSNE in (E) showing averaged expression scores of lineage classifiers.

We performed single-cell transcriptome analysis, using SMART-seq2 (Picelli et al., 2013) to achieve suitable sequencing depth and facilitate reliable comparison with human embryo scRNA-seq data. To avoid over-representation of trophectoderm cells, we manually excised most of the abembryonic region (mural trophectoderm) prior to dissociation. After quality control we analyzed transcriptomes from 159 day 3 and 108 day 4 blastoid cells.

t-distributed stochastic neighbor embedding (tSNE) revealed largely overlapping distributions on day 3 and day 4 (Figure 2A). Using exclusive lineage markers from the human E6/7 blastocyst (Guo et al., 2021; Petropoulos et al., 2016; Stirparo et al., 2018), almost all cells were assigned trophectoderm, epiblast, or hypoblast identities (Figures 2B and S2A). A small cluster of six cells (5 from day 3, 1 from day 4) was indeterminate between epiblast and hypoblast (Figure S2A). Averaged expression of classifier genes (Table S1) substantiated the lineage demarcations (Figure 2B). Illustrative marker gene profiles are shown in Figure 2C. Within the trophectoderm cluster, GATA2 and TEAD3 were uniformly high while early markers *CDX2* (Niakan and Eggan, 2013) and *SLC12A3* showed heterogeneous expression and were more prominent on day 3 than day 4. Naive status of the epiblast cluster was evidenced by *KLF17* and *ARGFX*. *PDGFRa*, which discriminates hypoblast from definitive endoderm, authenticated the hypoblast cluster.

We also sequenced cells from E5, E6, and E7 human embryos, bisected to reduce the mural trophectoderm. Consistent with previous analyses (Blakeley et al., 2015; Petropoulos et al., 2016; Stirparo et al., 2018), tSNE separated early (E5) from late (E6/7) blastocyst stages (Figures 2D and S2B). Expression of lineage classifiers identified ICM, trophectoderm, epiblast, and hypoblast clusters (Figure S2B).

We performed integrated tSNE analysis on blastoid and embryo data (Figures 2E and S2C). With few exceptions, identities independently determined for the blastoid and embryo datasets aligned in the integrated plot (Figure 2F). That is, almost all blastoid cells clustered together with embryo cells assigned to the same lineage. The few misaligned cells mostly originated from the embryos. Blastoid cells clustered more densely with E6/7 embryo cells (Figure 2E), consistent with late blastocyst similarity. However, the small group of intermediate cells in the blastoid samples were most related to E5 embryo ICM. *CDX2*, *SLC12A3*, and *GPRC5A* were expressed in most E5 trophectoderm cells but were heterogenous in E6 and E7 samples (Figure S2E). These observations suggest that blastoids may transit through a stage with features of the E5 blastocyst.

Overall, transcriptome analyses show a high degree of fidelity in lineage segregation between our blastoids and human blastocysts. Trophectoderm, authenticated by multiple markers, is well represented and there is no indication of abundant mismatched cells.

We inspected scRNA-seq data from recent reports of human blastoids (Liu et al., 2021; Yu et al., 2021) (Figure S3). Trophectoderm is by far the major cell type in the expanded human blastocyst. However, tSNE analysis of data from Yu et al. (2021) showed only a minor population of trophectoderm-related cells with a preponderance of EPI-related cells (Figure S3A). Two distinct clusters expressed *OCT4*, *SOX2*, and *NANOG* but differed in expression of many genes including the embryo naive epiblast marker *ETV4* (Figure S3B). Together they comprised more than 80% of cells, indicating a massive failure of pluripotent cells to differentiate or to acquire a consistent embryonic identity. Furthermore, the trophectoderm-like cluster entirely lacked expression of the general trophoblast lineage marker *TP63*. Blastoids created by reprogramming (Liu et al., 2021) contained a majority of cells with ambiguous identities (Figures S3C and S3D).

## Discussion

Availability of a reliable human blastocyst model will enable molecular and genetic dissection of early human embryogenesis, allow systematic exploration of culture conditions for assisted reproduction, and open a path for research into peri- and early post-implantation development. Our findings demonstrate that the innate lineage plasticity of human early epiblast and naive stem cells (Guo et al., 2021; Io et al., 2021) can be efficiently channelled into formation of entities with strong resemblance to the natural human blastocyst. Crucially, blastoid cells exhibited topologically restricted and mutually exclusive expression of markers of the three lineages, while scRNA-seq analysis confirmed the assignation of almost all blastoid cells to blastocyst stage trophectoderm, hypoblast, or epiblast.

Ability to form an authentic blastocyst-like entity solely from naive stem cells is a marked difference between human and mouse. Mouse embryonic stem cells are developmentally restricted from forming trophectoderm and blastoids are therefore created by combining cells of different lineages (Posfai et al., 2021; Rivron et al., 2018). In contrast, human naive stem cells can readily be induced into trophectoderm. In cell clusters, the trophectoderm cells form an outer epithelial monolayer that encloses persisting undifferentiated naive cells and initiates polarized ion transport. The resulting fluid uptake leads to formation of an expanded cavity. Induction of trophectoderm with PD+A83 should be limited in duration to preserve a naive inner cell population and allow hypoblast differentiation. Other epithelia can form cystic structures and cavitation alone is not sufficient to assert trophectoderm identity. The *GATA3:mKO2* reporter, which is highly expressed in all trophectoderm cells, provides reassurance.

Blastoid formation results in an entity with high similarity to the human blastocyst developed after *in vitro* fertilization. Interestingly, however, the naive epiblast starting stage for blastoids is distinct from the embryological origin, compacted morula. Therefore, the initial differentiation and morphokinetic pathways are dissimilar. Nonetheless, a three-dimensional structure of appropriate size and shape is formed in which the three lineages are correctly specified and segregated. This outcome implies a flexible coupling between differentiation and morphogenesis. Understanding how structures with equivalent topology and composition are generated from different embarkation points will shed light into the resilience of tissue formation.

Overall, our findings demonstrate the capability for generation of human blastoids at scale in simple and defined conditions. The blastoids fulfil several key criteria for a useful model of the human blastocyst: (1) correct topological segregation of lineages evidenced by appropriately localized and mutually exclusive expression of multiple marker proteins; (2) clustering of scRNA-seq data into three unambiguous lineages; (3) high transcriptome fidelity with the human embryo, with few or no unassigned cells; (4) single epiblast population with naive features; (5) timescale of morphogenesis similar to human blastocyst formation (3–4 days); (6) coordinated progression of morphogenesis and lineage segregation; (7) robust and scalable procedure with a high yield of cavitated tri-lineage structures (>80%); and (8) consistency across multiple stem cell lines.

### Limitations of the study

Differentiation dynamics vary slightly between human naive stem cell lines. For high efficiency, the cell seeding number and duration of PD+A83 treatment may need fine-tuning for individual lines. Microfluidics and bioengineering approaches (Zheng et al., 2019) may yield a more standardized procedure. We also observe that hypoblast cell number varies and is not proportionate to the number of epiblast cells in blastoids. However, the presentation of hypoblast cells in the human embryo is variable. The blastoid system provides an opportunity to delineate stimuli and mechanisms that regulate specification and sorting of hypoblast in human embryos. Gene expression is similar but not identical between blastoids and blastocysts and this may also be a fruitful area for investigation. Finally, the present study focuses on characterization of a blastocyst-stage entity with only preliminary analysis of further developmental potential. A future prospect is to use the blastoid model to develop improved culture conditions that reliably mimic peri- and early post-implantation embryogenesis. This will require specific, objective, and quantifiable measures of morphogenesis and differentiation, ideally combined with functional assays. Better systems for extended culture can then be applied to human embryos for direct comparison of developmental trajectories and potential.

## STAR★Methods

### Key resources table

**Table:** 

REAGENT or RESOURCE	SOURCE	IDENTIFIER
**Antibodies**		

Rabbit polyclonal anti-KLF17	Atlas Antibodies	Cat# HPA024629, RRID:AB_1668927
Goat polyclonal anti-NANOG	R&D System	Cat# AF1997, RRID:AB_355097
Rabbit polyclonal anti-NANOG	Abcam	Cat# ab21624, RRID:AB_446437
Rabbit polyclonal anti-Oct-4	Cell Signaling Technology	Cat# 2750, RRID:AB_823583
Mouse monoclonal anti-Sox2	Santa Cruz	Cat# sc-365823, RRID:AB_10842165
Rat monoclonal anti-Gata-4	Thermo Fisher Scientific	Cat# 14-9980-82, RRID:AB_763541
Goat polyclonal anti-SOX17	R&D System	Cat# AF1924, RRID:AB_355060
Goat polyclonal anti-Otx2	R&D System	Cat# AF1979, RRID:AB_2157172
Mouse monoclonal anti-Gata3	Thermo Fisher Scientific	Cat# MA1-028, RRID:AB_2536713
Mouse monoclonal anti-AP2 gamma	Santa Cruz	Cat# sc-12762, RRID:AB_667770
Rabbit monoclonal anti-CK7	Abcam	Cat# ab181598, RRID:AB_2783822
Mouse monoclonal anti-CK18	Abcam	Cat# ab668, RRID:AB_305647
Rabbit monoclonal anti-CK18	Abcam	Cat# ab133263, RRID:AB_11155542
Goat polyclonal anti-Lamin B	Santa Cruz	Cat# sc-6217, RRID:AB_648158
Donkey anti-Goat Alexa Fluor 405	Abcam	Cat# ab175664, RRID:AB_2313502
Donkey anti-Goat Alexa Fluor 488	Thermo Fisher Scientific	Cat# A32814, RRID:AB_2762838
Donkey anti-Rabbit Alexa Fluor 488	Thermo Fisher Scientific	Cat# A32790, RRID:AB_2762833
Donkey anti-Rat Alexa Fluor 488	Thermo Fisher Scientific	Cat# A-21208, RRID:AB_2535794
Donkey anti-Goat Alexa Fluor 555	Thermo Fisher Scientific	Cat# A-21432, RRID:AB_2535853
Donkey anti-Rabbit Alexa Fluor 555	Thermo Fisher Scientific	Cat# A-31572, RRID:AB_162543
Donkey anti-Mouse Alexa Fluor 555	Thermo Fisher Scientific	Cat# A-31570, RRID:AB_2536180
Donkey anti-Goat Alexa Fluor 647	Thermo Fisher Scientific	Cat# A-21447, RRID:AB_2535864
Donkey anti- Rabbit Alexa Fluor 647	Thermo Fisher Scientific	Cat# A-31573, RRID:AB_2536183
Donkey anti- Mouse Alexa Fluor 647	Thermo Fisher Scientific	Cat# A-31571, RRID:AB_162542
Rabbit monoclonal anti-CK7	Abcam	Cat# ab181598 RRID:AB_2783822
Mouse monoclonal anti-hCGB	Abcam	Cat# ab9582 RRID:AB_296507
Mouse monoclonal anti-Podocalyxin	R&D System	Cat# MAB1658, RRID:AB_2165984

**Chemicals, peptides, and recombinant proteins**		

MEK inhibitor PD0325901	ABCR	Cat#AB 253775
Tankyrase inhibitor XAV939	Cell Guidance Systems	Cat#SMS38-200
aPKC inhibitor Gö6983	Bio-Techne	Cat#2285
Rho associated kinase inhibitor Y-27632	Merck Chemicals	Cat#688000-100MG
Human leukemia inhibitory factor (LIF)	Made in-house	N/A
Activin/nodal receptor inhibitor A83-01	Generon	Cat#A12358-50

**Complete Culture Media and Cell Dissociation Reagent**		

N2B27	Made in-house	N/A
Accutase	Millipore	Cat#SCR005
TrypLE™ Express Enzyme	Thermo Fisher Scientific	Cat#12605028

**Cell Attachment Proteins and Peptides**		

Geltrex	Thermo Fisher Scientific	Cat#A1413302

**Deposited data**		

scRNaseq	This study	GSE171820
scRNaseq	(Petropoulos et al., 2016)	E-MTAB-3929
scRNaseq	(Yu et al., 2021)	GSE150578
scRNaseq	(Liu et al., 2021)	GSE156596

**Experimental models: cell lines**		

HNES1-*GATA3:mKO2*	(Guo et al., 2021)	N/A
niPSC HDF75	(Bredenkamp et al., 2019b)	N/A
cR-Shef6	(Guo et al., 2017)	N/A

**Software and algorithms**		

STAR v2.7.7a	(Dobin et al., 2013)	https://github.com/alexdobin/STAR
featureCounts (Subread v2.0.1)	(Liao et al., 2014)	http://subread.sourceforge.net/
Rtsne v0.15		https://github.com/jkrijthe/Rtsne
Harmony v1.0	(Korsunsky et al., 2019)	https://github.com/immunogenomics/harmony
Seurat v4.0	(Stuart et al., 2019)	https://satijalab.org/seurat/
Trim-Galore! v0.6.6		https://www.bioinformatics.babraham.ac.uk/projects/trim_galore/
R v4.0.3		https://www.r-project.org/

### Resource availability

#### Lead contact

Further information and requests for resources and reagents should be directed to and will be fulfilled by the Lead Contact, Ge Guo, g.guo@exeter.ac.uk

#### Materials availability

All stable reagents generated in this study are available from the Lead Contact without restriction except for human embryo-derived cell lines, for which permission must be requested from the UK Stem Cell Steering Committee and a Materials Transfer Agreement completed.

#### Data and code availability

The RNaseq data generated in this paper are deposited in Gene Expression Omnibus with accession code GSE171820

### Experimental model and subject details

#### Human embryos

The use of supernumerary human embryos in this research is approved by the Multi-Centre Research Ethics Committee, approval O4/MRE03/44, and licensed by the Human Embryology & Fertilization Authority of the United Kingdom, research license R0178.

#### Cell culture

Cell lines are listed in the Key Resources Table. Cellswere cultured in humidified incubators at 37þC in 7% CO2 and 5% O2. Cells were cultured without antibiotics and tested negative for mycoplasma by periodic PCR screening.

### Method details

#### Human naive pluripotent stem cell culture

Human naive pluripotent stem cells were propagated in PXGL medium as described (Bredenkamp et al., 2019a). PXGL comprises 1 μM PD0325901 (P), 2 μM XAV939 (X), 2 μM Gö6983 (G) and 10 ng/mL human LIF (L) in N2B27 basal medium. Cells were cultured on irradiated MEF feeders. Rho associate kinase inhibitor (Y-27632) and Geltrex (0.5μL per cm^2^ surface area, Thermo Fisher Scientific, A1413302,) were added to media during replating. Cells were passaged by dissociation with Accutase (Biolegend, 423201) every 3-4 days.

#### Generation of blastoids

PXGL cultures of naive stem cells in exponential growth were dissociated with TrypLE for 5 min and pelleted in washing medium (DMEM/F12 supplemented with 0.1% BSA). The pellet was resuspended in PD+A83+Y medium (N2B27 supplemented with 1.5 μM PD0325901, 1 μM A83-01 and 10 μM Y-27632). Cell number was counted using a hematocytometer and cell density was adjusted to 1000 cell per ml. 50-200 cells were dispensed into each well of an ultra-low attachment multiple-well plate (Corning Coster) with a multichannel pipette. The plates were centrifuged at 300 g for 4 min at room temperature to cluster cells at the bottom of the wells. After 42-48 h, cell aggregates were manually transferred into a non-adherent, ‘U’-bottomed 96-well (Greiner) containing pre-warmed N2B27 supplemented with 0.5 μM A83-01) using a mouth-controlled pipette. At the end of day 3, cysts were manually transferred into N2B27 medium without either inhibitor.

#### Attachment culture of blastoids

Day 4 blastoids were moved manually from a multiwell plate to a μ-Slide 8-well (ibidi) coated with geltrex and cultured in N2B27 medium. Half of the medium was replaced every day. Blastoids usually attached to the plate and began to outgrow within two days. Cultures were fixed for staining after 4 days of attachment culture

#### Human embryos

Supernumerary frozen blastocysts (E5 or E6) were thawed and cultured in N2B27 medium under mineral oil. Embryos that were well-expanded after thawing were judged to be E6 and processed immediately, while the majority of embryos were cultured for 24 h for development to expanded blastocysts (E6 or E7). The zona pellucida was removed using acid Tyrode’s solution.

#### Immunostaining

Blastoids, outgrowths and embryos were fixed with 4% PFA in PBS for 15 min at room temperature. Samples were rinsed in PBS containing 3 mg/mL polyvinylpyrrolidone (PBS/PVP) and permeabilized with PBS/PVP containing 0.25% Triton X-100 for 30 min. Blocking was performed in embryo blocking buffer comprising PBS supplemented with 0.1% BSA, 0.01% Tween20 and 2% donkey serum for 2-3 h at 4°C. Samples were incubated in the blocking buffer with or without 500 ng/mL DAPI for 1 h at room temperature in the dark. DAPI-stained samples were rinsed three times for 15 min in blocking buffer. The staining process for blastoids and embryos was performed in microwell miniTrays.

#### Imaging

Blastoids and embryos were transferred in small drops of blocking buffer onto poly-D-lysine-coated glass-bottom dishes under mineral oil or μ-slide 18 well-Flat and imaged using a Leica TCS SP5 confocal microscope. Images were processed using Fiji. Widefield images were taken using Leica DMI4000 or DMI8. Numbers of GATA4+ or KLF17+ cells were counted manually from the confocal images. The diameter of each blastoid was measured using Fiji.

#### Transcriptome sequencing

Embryos and blastoids were placed singly in drops of M2 medium and the mural trophectoderm (mTE) was manually excised using finely drawn glass needles. mTE fragments were transferred to drops of Accutase and the ICM plus polar trophectoderm samples were transferred to TrypLE using glass capillaries or micropipettes. After incubation for 7-10 min at 37°C for Accutase or room temperature for TrypLE. Samples were transferred to drops of filtered M2 and dissociated by trituration using a finely drawn glass capillary with diameter just larger than a single cell. Single cells were transferred into individual wells of a 96-well plate containing Smart-seq2 single-cell lysis buffer and immediately frozen. Smart-seq2 libraries were prepared as described (Picelli et al., 2013) and sequenced using the Illumina Novaseq platform in paired end format.

### Quantification and statistical analysis

#### RNA-Seq data processing

Adaptor sequences and low-quality base calls were trimmed using Trim Galore! v0.6.6 with default parameters. Trimmed reads were aligned to the Human reference genome (GRCh38) using STAR v2.7.7a (Dobin et al., 2013) in paired-end mode with default parameters. Gene level counts were generated using FeatureCounts from Subread v2.0.1 (Liao et al., 2014).

#### Transcriptome analysis

Cells with detection of fewer than 6000 expressed genes were discarded and the remaining samples were log_2_FPKM normalized using custom scripts. Only protein coding genes were considered in the analyses. tSNE (t-distributed stochastic neighbor embedding) plots were computed using variable genes across the dataset under analysis with Rtsne v0.15 (M. Krijthe, 2015) and visualized using ggplot2 in R v4.0.3. The AddModuleScore function from Seurat v4.0 (Stuart et al., 2019) was used to score and color cells according to the expression of signature gene lists for different cell lineages derived from (Petropoulos et al., 2016; Stirparo et al., 2018). To obtain the signature gene list, FPKM values were used to compute Pearson’s correlation coefficients with a binary feature vector for each cell population consisting of values of 1 for the considered cell type and 0 for other cell types. Genes with a correlation coefficient of greater than 0.4 were used as lineage classifiers (Table S1).

Harmony v1.0 (Korsunsky et al., 2019) was used for batch correction between the embryo and blastoid datasets using the first 50 principle components produced via principal component analysis.

## Supplemental information

Document S1. Figures S1–S3

Table S1. Lineage-classifier genes extracted from (Petropoulos et al., 2016; Stirparo et al., 2018), related to Figure 2

Document S2. Article plus supplemental information
