# Supplementary material for: Naive stem cell blastocyst model captures human embryo lineage segregation
Source: Cell Stem Cell. 2021 Jun 3;28(6):1016–1022.e4. doi: 10.1016/j.stem.2021.04.031 (PMC8189436; doi:10.1016/j.stem.2021.04.031)
Supplement: Document S1. Figures S1–S3 [file mmc1.pdf]

Cell Stem Cell, Volume *28*

Supplemental Information

Naive stem cell blastocyst model captures human embryo lineage segregation

Ayaka Yanagida, Daniel Spindlow, Jennifer Nichols, Anish Dattani, Austin Smith, and Ge Guo

SUPPLEMENTAL INFORMATION INDEX

Figure S1. Formation of human blastocyst models (blastoids), relates to Figure 1
Figure S2. Single cell transcriptome analyses, relates to Figure 2
Figure S3. Single cell transcriptome analysis of previously reported blastoids, relates to Figure2

SUPPLEMENTAL LEGENDS

Figure S1. Formation of model human blastocysts (blastoids), relates to Figure 1

A. Images of day 3 HNES1-*GATA3:mKO2* cysts formed from initial cell numbers of 100 or 125. Images were taken from a continuous area of a 96 well plate to represent the range of morphologies.
B. Images of disorganized, aggregate, single cyst or multiple cyst structures on day 3.
C. Quantification of structures on day 3.
D. Images of day 3 cysts with no ICM (left, GATA3+), no hypoblast (middle, GATA3+, KLF17+), and with three lineages (right, KLF17+, GATA4+, GATA3+).
E. Pie chart of lineage marker expression in day 3 blastoids.
F. Quantification of the number of KLF17 or GATA4 positive inner cells in blastoids.
G. Images of day 3 niPSC_HDF75 blastoids in contiguous wells. Initial cell number seeded was 150.
H. Immunofluorescence staining for OTX2 in day 3 HNES1mKO2 blastoid.
I. Z-projections of day 3 blastoids formed from reset cR-Shef6 naïve cells
J. Z-projections of day 4 blastoids formed from niPSC_HDF75 naïve cells. All scale bars are 50 μm.

Figure S2. Single cell transcriptome analysis, relates to Figure 2

A. t-distributed stochastic neighbour embedding (tSNE) analysis of blastoids coloured to show marker-based cell lineage assignment. EPI, epiblast; Hyp, hypoblast; TE, trophectoderm; intermediate, cells without clear signature
B. tSNE analysis of human embryos.
C. tSNE plot in B coloured to show averaged expression scores of gene sets enriched in human embryo lineages (Petropoulos et al., Cell, 2016): EPI, epiblast (E6/7); Hyp hypoblast (E6/7); TE, trophectoderm (E6/7); ICM (E5).
D. Integrated tSNE of blastoids and embryos
E. Expression (FPKM) of selected lineage markers in integrated tSNE plots.

Figure S3. Single cell transcriptome analysis, relates to Figure 2

scRNA-seq data from (Yu et al., 2021) and (Liu et al., 2021) were downloaded and analysed as in Figure 2 and Figure S2.

A. tSNE analysis of day 9 blastoid data from Yu et al. (2021) coloured to show averaged expression scores of embryo lineage classifiers.
B. Expression (FPKM) of selected lineage markers in samples from Yu et al. (2021).
C. tSNE analysis of induced blastoid data from Liu et al. (2021) coloured to show averaged expression scores of embryo lineage classifiers.
D. Expression (FPKM) of selected lineage markers in samples from Liu et al. (2021)

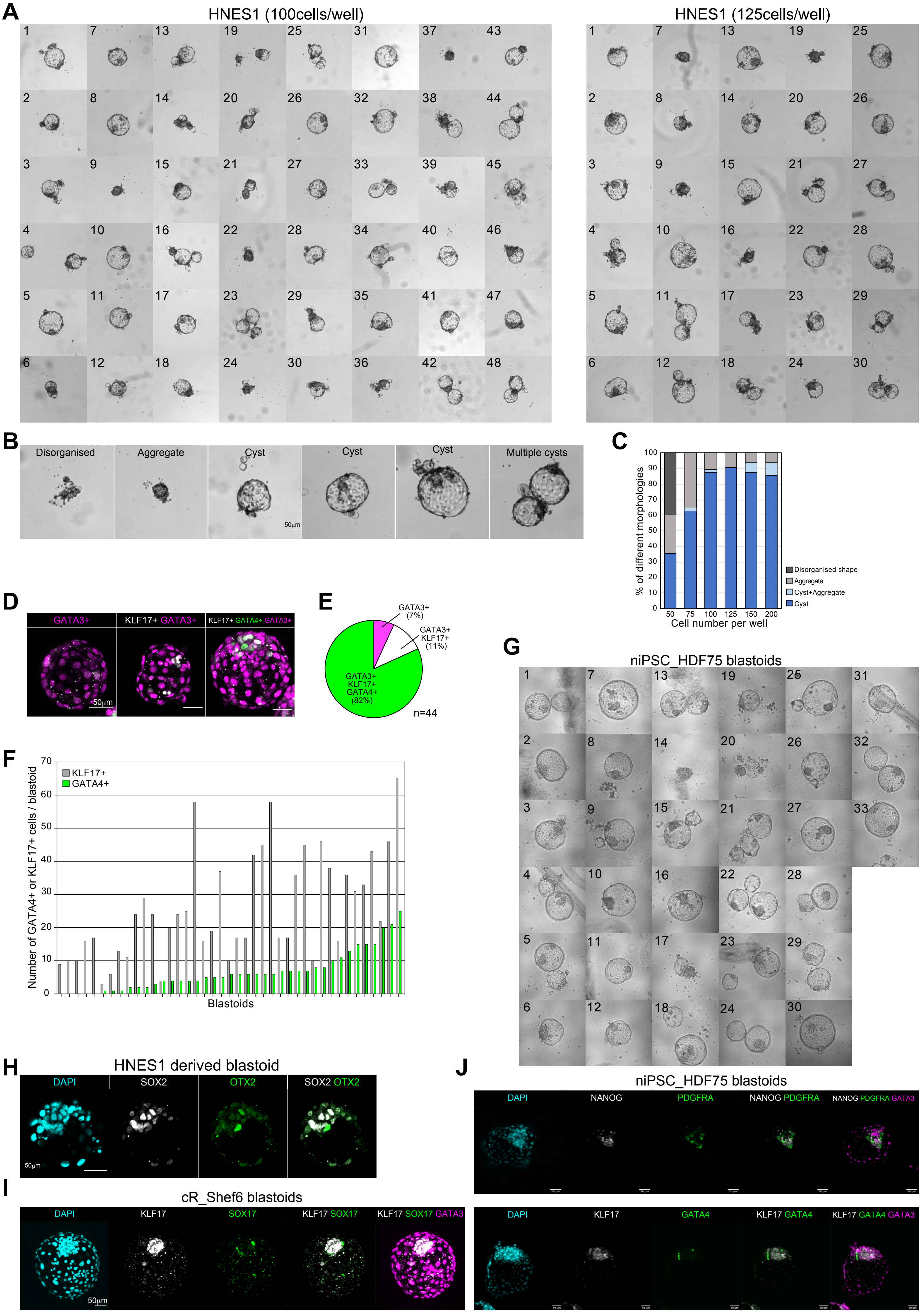
A
HNES1 (100cells/well)
HNES1 (125cells/well)
B
Disorganised
Aggregate
Cyst
Cyst
Cyst
Multiple cysts
50μm
C
% of different morphologies
Cell number per well
50 75 100 125 150 200
Disorganised shape
Aggregate
Cyst+Aggregate
Cyst
D
GATA3+
KLF17+ GATA3+
KLF17+ GATA4+ GATA3+
50μm
E
GATA3+ (7%)
GATA3+ KLF17+ (11%)
GATA3+ KLF17+ GATA4+ (82%)
n=44
F
KLF17+
GATA4+
Number of GATA4+ or KLF17+ cells / blastoid
Blastoids
G
niPSC_HDF75 blastoids
H
HNES1 derived blastoid
DAPI
SOX2
OTX2
SOX2 OTX2
50μm
I
cR_Shef6 blastoids
DAPI
KLF17
SOX17
KLF17 SOX17
KLF17 SOX17 GATA3
50μm
J
niPSC_HDF75 blastoids
DAPI
NANOG
PDGFRA
NANOG PDGFRA
NANOG PDGFRA GATA3
DAPI
KLF17
GATA4
KLF17 GATA4
KLF17 GATA4 GATA3

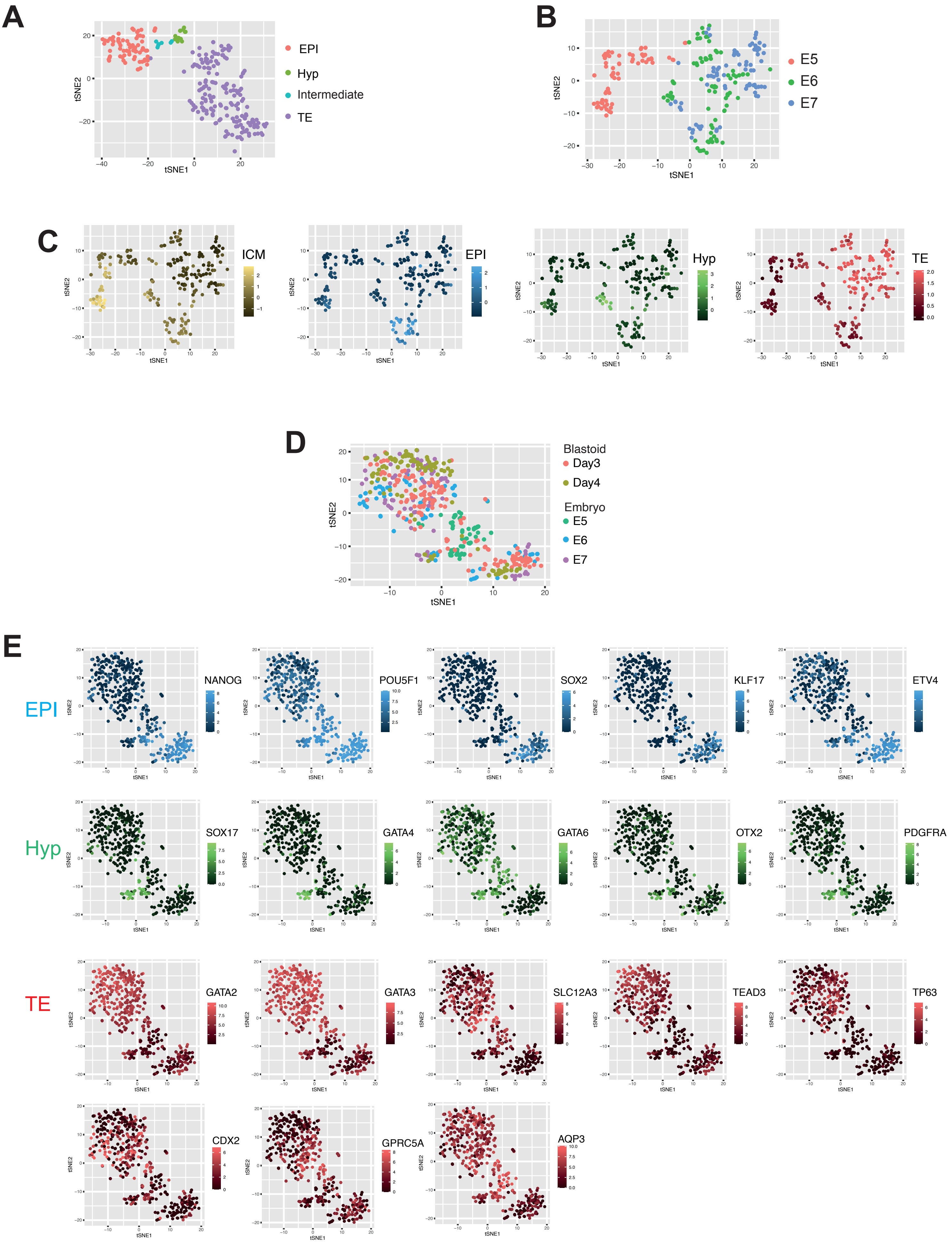
A
EPI
Hyp
Intermediate
TE
tSNE1
tSNE2
B
E5
E6
E7
C
ICM
EPI
Hyp
TE
D
Blastoid
Day3
Day4
Embryo
E5
E6
E7
E
EPI
NANOG
POU5F1
SOX2
KLF17
ETV4
Hyp
SOX17
GATA4
GATA6
OTX2
PDGFRA
TE
GATA2
GATA3
SLC12A3
TEAD3
TP63
CDX2
GPRC5A
AQP3

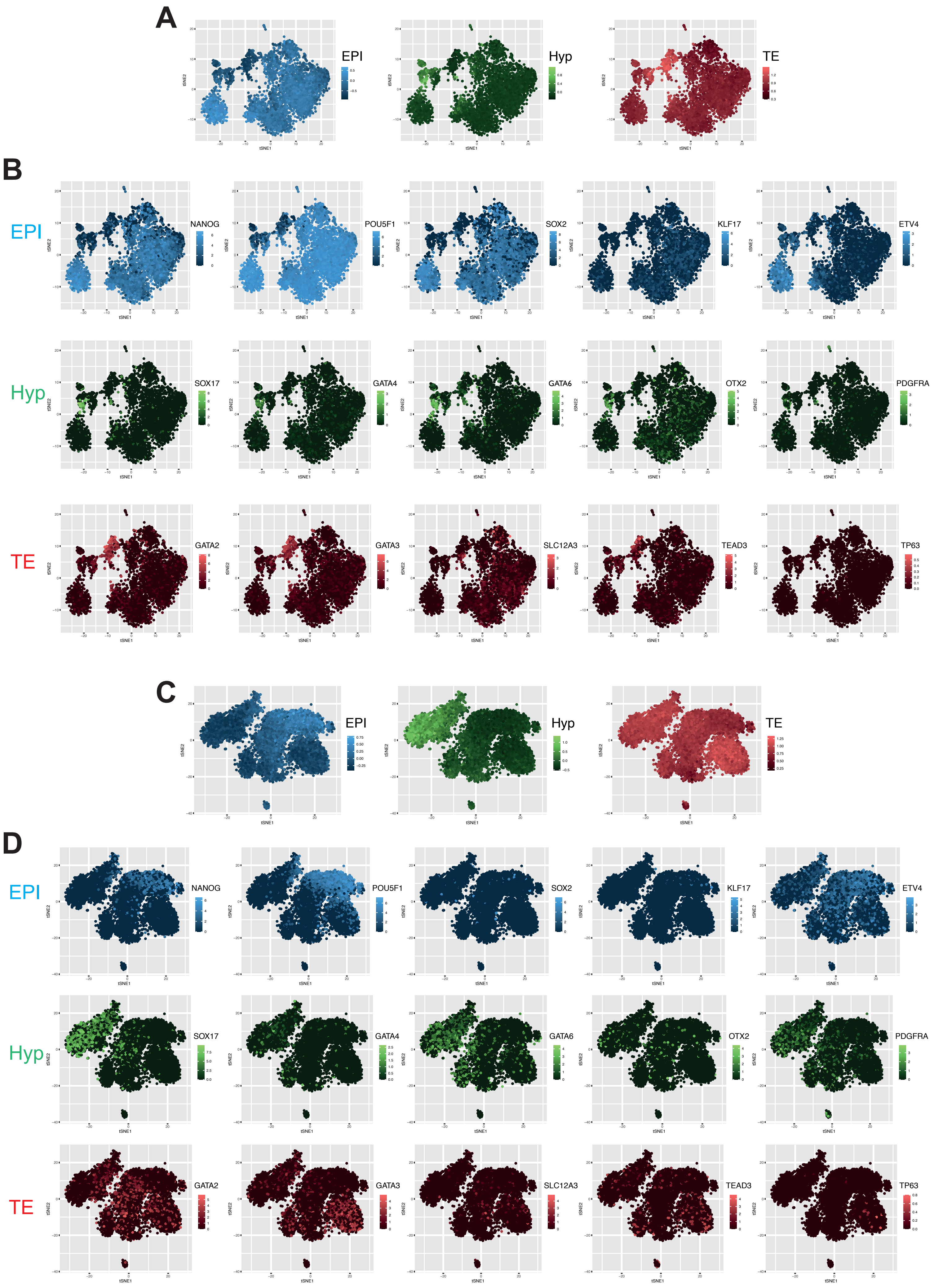
A
EPI
Hyp
TE
tSNE1
tSNE2
B
EPI
NANOG
POU5F1
SOX2
KLF17
ETV4
Hyp
SOX17
GATA4
GATA6
OTX2
PDGFRA
TE
GATA2
GATA3
SLC12A3
TEAD3
TP63
C
EPI
Hyp
TE
D
EPI
NANOG
POU5F1
SOX2
KLF17
ETV4
Hyp
SOX17
GATA4
GATA6
OTX2
PDGFRA
TE
GATA2
GATA3
SLC12A3
TEAD3
TP63
